# Targeting BRAF-mutant tumours with TGFBR1 inhibitors

**DOI:** 10.18632/aging.101169

**Published:** 2017-01-31

**Authors:** Lindsay C Spender, Gareth J Inman

**Affiliations:** Division of Cancer Research, School of Medicine, University of Dundee, Dundee, UK

**Keywords:** TGFβ, BRAF, melanoma, therapy

Transforming growth factor beta (TGFβ) is a major contributing factor to many diseases including progressive cancers [1]. Given the normal homeostatic functions of TGFβ in controlling cell proliferation, immune surveillance and angiogenesis, however, it is much less clear which cancer patients would derive clinical benefit from the administration of anti-TGFβ targeted therapy. The use of these therapies is complicated not only by toxicity issues, but also by concerns that interfering with tumour suppressive functions of TGFβ may ultimately exacerbate disease. Thus, identifying when and where TGFβ signalling promotes tumour progression [2] is key to the success of numerous approaches to block TGFβ signalling using a range of humanised antibodies, small molecule inhibitors (SMIs) and antisense oligonucleotides currently being evaluated in clinical trials [3]. Our recent data now provides insight into when these therapies might succeed [4].

To investigate when anti-TGFβ targeted therapies might be used appropriately, we screened cancer cell lines carrying defined cancer-associated genetic abnormalities in the RAS/RAF/MEK/ERK signalling pathway for sensitivity to SMIs of the TGFβ type 1 receptor (TGFBR1). In assays specifically designed to measure cancer-stem cell like properties including clonogenicity and anchorage independent growth assays, we found that cells carrying mutant BRAF were dependent on TGFβ signalling for growth. Although cells carrying mutant KRAS (n=11), NRAS (n=4) and BRAF (n=7) all exhibit constitutive activation of the MAPK pathway, only mutant BRAF cells were universally inhibited by the TGFBR1 inhibitor. Our data were confirmed by siRNA knockdown of the receptor to ensure that the effects of the inhibitor were on-target. Additionally, the dependence of mutant BRAF cells on secreted autocrine TGFβ signalling for formation of xenograft tumours was demonstrated by stably expressing short-hairpin shRNA targeting TGFβ RNA in the cancer cells. The ability of these cells to establish tumours in mice was significantly impaired by TGFβ knockdown. The data suggests, therefore, that mutant BRAF cells are ‘hard-wired’ to depend on autocrine TGFβ signalling for growth in stressful conditions. The data also implies that the presence of mutant BRAF in sequenced tumour biopsies could act as a biomarker for stratifying patients for anti-TGFβ therapy. The mechanism of TGFβ-mediated tumour cell growth promotion remains elusive, but appears to be independent of canonical TGFβ signalling pathways involving the SMAD transcription factors and may involve activation of the small GTPase RHO-A. It might be misleading, therefore, to use the phosphorylation of SMADs downstream of TGFβ receptor activation as an additional biomarker in tumour tissue biopsies to indicate the potential for TGFBR1 inhibitor use in cancer.

The patients most likely to benefit include melanoma cancer patients since mutation of BRAF is detected in approximately 50% of melanomas [5]. Melanoma is particularly prevalent, and mortality highest, in the aging population with more than 80% of deaths from melanoma occurring in people aged over 50 (www.cancerresearch.org). The discovery of the genetic abnormalities associated with melanoma has resulted in an astonishing effort from researchers and the pharmaceutical industry alike to develop and test small molecule inhibitors of mutant BRAF (BRAFi). While these drugs (e.g. vemurafenib) have revolutionised treatment and increased patient survival, chemo-resistance remains a significant clinical issue and the majority of patients relapse and die from drug-resistant metastatic disease. Our analysis of the effect of TGFβ inhibitors in both drug-naïve and vemurafenib-resistant patient derived cells therefore provides valuable information relating to when and where TGFβ inhibitors might be effective.

We found that TGFBR1 inhibitors remained effective against vemurafenib-resistant patient derived cells. Furthermore, TGFβ inhibitors prevented the enhanced cell growth caused by paradoxical activation of the MAPK pathway seen in cells treated with BRAFi. Taken together targeting TGFβ signalling in mutant BRAF melanoma is predicted to inhibit tumour growth even in drug-resistant disease, however, the tumour cell microenvironment appears to impact significantly on inhibitor efficacy. We found that cell density reduced the ability of TGFBR1 inhibitors to prevent melanoma growth. In these less stressful conditions, melanoma cells were not dependent on TGFβ signalling to the same extent. The addiction melanoma cells have to TGFβ signalling correlates with isolation and anchorage independence - situations encountered during tumour spread and outgrowth of metastasis. Using zebrafish xenograft models of melanoma cell metastasis we were able to track single fluorescently tagged cells during the process of migration into tail-fin tissue. As predicted, TGFBR1 inhibitors and TGFBR1 shRNA both reduced melanoma cell migration. In conclusion we envisage that TGFBR1 inhibitors are more likely to be effective in targeting cancer stem cell like properties and metastatic outgrowth, rather than in reducing the size of established tumours and the experience, so far, of investigators working on the clinical development of the TGFBR1 inhibitors supports this idea [6]. To maximise the clinical potential of TGFBR1 inhibitors, therefore, we propose that investigations should focus on their use in the prevention of metastatic recurrence after reducing the established tumour burden by conventional surgical and chemotherapeutic approaches.

## References

[1] Blobe GC (2000). N Engl J Med..

[2] Inman GJ (2011). Curr Opin Genet Dev.

[3] Akhurst RJ, Hata A (2012). Nat Rev Drug Discov.

[4] Spender LC (2016). Oncotarget.

[5] Davies H (2002). Nature.

[6] Herbertz S (2015). Drug Des Devel Ther..

